# Multisensory suppressive neurons can implement Bayesian-like nonlinearly weighted sensory combination

**DOI:** 10.3389/fnhum.2026.1676655

**Published:** 2026-07-23

**Authors:** Vincent A. Billock, Kacie Dougherty, Adam M. Preston, M. Alex Meredith

**Affiliations:** 1 Leidos, Inc., at the Naval Aerospace Medical Research Laboratory, NAMRU-D, Wright-Patterson AFB, OH, United States; 2 Princeton Neuroscience Institute, Princeton University, Princeton, NJ, United States; 3 Naval Aerospace Medical Research Laboratory, NAMRU-D, Wright-Patterson AFB, OH, United States; 4 Department of Anatomy and Neurobiology, Virginia Commonwealth University, Richmond, VA, United States

**Keywords:** Bayesian integration, maximum likelihood estimation, multisensory neural modeling, Poisson neural firing rate statistics, Schrödinger model, suppressive multisensory neurons

## Abstract

**Introduction:**

We address two related questions in multisensory integration: What are suppressive multisensory and multimodal neurons good for, and how could neurons implement the earliest stages of weighted cue averaging often found in multisensory integration psychophysics? Mildly suppressive multisensory and binocular neurons are ideally positioned to implement weighted averaging. Of the many possible weighted averages, the two most interesting theoretical possibilities are Maximum Likelihood Estimation (MLE) – a Bayesian methodology – and nonlinear magnitude weighting, posited by Erwin Schrödinger for binocular averaging. The MLE approach weights reliability (expressed as inverse relative variance), which is trickier to implement in neurons than nonlinear magnitude weighting.

**Methods and Results:**

We tested these two models on three classes of cortical multisensory neurons. We find that suppressive audio-visual, visual-tactile and audio-tactile neurons are well positioned to implement a weighted average of their two inputs, a result consistent with our prior findings in binocular suppressive neurons. The actual neural firing rates more closely resemble Schrödinger’s nonlinear weighted average, but the output of this Schrödinger model is well correlated with the output of a rigorously implemented MLE reliability-weighted average.

**Discussion:**

One possible interpretation is that evolution pushed sensory integration towards a Bayesian-like outcome and settled – at least at an early neural level – for a nonlinear approximation that was easier than inverse-relative-variance-weighting to implement in neural systems.

## 1 Introduction

There are two related questions in multisensory neuroscience that can be addressed by tackling both simultaneously. One question is: how could neural correlates of Bayesian-like information combination be implemented? Bayesian ideas have strongly influenced sensory research in general and multisensory integration in particular (Young et al., 1993; Ernst and Banks, 2002; Alais and Burr, 2004; Ernst, 2012; Angelaki et al., 2012). For example, Ernst and Banks (2002) modeled human visual-tactile cue combination and found that a Bayesian-like approach—Maximum Likelihood Estimation (MLE) performed well. Similarly, Alais and Burr (2004) found that MLE predicted audio-visual information combination in the ventriloquism effect. There are also data that support an MLE model for visual-vestibular integration and for other systems (Angelaki et al., 2012; Morgan et al., 2008; Butler et al., 2010; Fetsch et al., 2012). The MLE model for combining two information channels is a weighted average with the weights determined by reliability, which is computed from inverse relative variance and can be derived from Bayesian considerations (Landy et al., 2011). Let *Rmode_1_* and *Rmode_2_* be firing rates in response to stimulation of *Mode_1_* and *Mode_2_*, and *σ_1_^2^ and σ_2_^2^* be the firing rate variances (Equations 1–4).


$$\text{MultiModalFiringRate}=k_{1}\text{Rmod}e_{1}+k_{2}\text{Rmod}e_{2}$$ (1)

where


$$k_{1}=(1/\sigma_{1}^{2})/(1/\sigma_{1}^{2}+1/\sigma_{2}^{2})=\sigma_{2}^{2}/(\sigma_{1}^{2}+\sigma_{2}^{2})$$ (2)

$$k_{2}=(1/\sigma_{2}^{2})/(1/\sigma_{1}^{2}+1/\sigma_{2}^{2})=\sigma_{1}^{2}/(\sigma_{1}^{2}+\sigma_{2}^{2})$$ (3)

which simplifies to


$$\text{MultiModalFiringRate}=(\begin{array}{l} \sigma_{2}^{2}\text{Rmod}e_{1}+\\ \sigma_{1}^{2}\text{Rmod}e_{2} \end{array})/(\sigma_{1}^{2}+\sigma_{2}^{2})$$ (4)

This model assumes Gaussian statistics, or cue independence, and can be derived from Bayesian theory by assuming flat priors or minimum combined variance (Young et al., 1993; Ernst and Banks, 2002; Alais and Burr, 2004; Landy et al., 2011), assumptions that may not always hold (Ernst, 2012; Angelaki et al., 2012; Meijer and Nopppeney, 2020; Scarfe, 2022). It is closely related to the Kalman filter used in engineering (Ernst and Bülthoff, 2004; Baker and Wade, 2017). This is an interesting and powerful model, but in principle it needs some neural mechanism to estimate/store the variances of the two channels, on flexible timescales set by particularities of psychophysical experiments, and it is not clear how to implement this in neurons, without relying on assumptions that are not well supported (but see Discussion for some special cases that may elide this problem). Moreover, there are psychophysical data on cue combinations that show significant deviations from the predictions of MLE. Some of these studies found that the channel weighting required deviate from the Bayesian prediction (Butler et al., 2010; Meijer et al., 2019; Oruc et al., 2003; Rahnev and Denison, 2018); some found alternatives to MLE that worked better for their data (Domini, 2022; Kemp et al., 2023); and Rosas et al. (2005, 2007) found that variance was not reduced in a manner consistent with MLE (see the Discussion for details.) This motivates us to entertain alternative and perhaps complementary models to MLE. This paper offers a simple alternative, which is not Bayesian-by-design (takes no account of system history or probabilities), but still responds similarly to (and in some aspects approximates) a Bayesian MLE system and could be implemented in individual neurons.

A second question in sensory neuroscience is: what do suppressive multisensory and multimodal cells do for perception? Suppressive multisensory cells were first described in rattlesnake visual-infrared interactions (Newman and Hartline, 1981, 1982). Rattlesnakes and other pit vipers see visible light using their eyes and see heat using their facial pits, whose pinhole-like optics image the world onto heat-sensitive demyelinated trigeminal neurons. Both systems project to optic tectum where there are some neurons that can fire for either visual or infrared information and enhance their firing rates when both signals are present, and other neurons that can fire for either visual or infrared information, but actually reduce their firing rates, relative to the best sensory response, if both signals are present. Newman and Hartline (1981, 1982) plausibly proposed that these suppressive snake neurons could be used to distinguish warm-blooded prey from cold-blooded prey; e.g., by comparing the responses of facilitatory and suppressive visual-infrared neurons to the same stimulus. Subsequent discoveries found suppressive cortical neurons in monkey, cat, and ferret, for visual–visual, audio-visual, visual-tactile and audio-tactile interactions (Miller et al., 1993; Barraclough et al., 2005; Meredith et al., 2012; Foxworthy et al., 2013, Meredith and Allman, 2015), but the rattlesnake discrimination argument is hard to extend to these systems and most multisensory neural studies have emphasized bimodal neurons with enhanced—not suppressive—responses. To understand suppressive multisensory neurons, it is useful to consider a closely analogous system which uses suppressive neurons in a non-Bayesian nonlinear weighted average: binocular vision. Information from the two eyes is combined in visual cortex. Near-threshold binocular vision is enhanced relative to monocular responses; on average binocular sensitivity is about 50% greater than the stronger monocular response. Binocular cells that enhance their firing rates to joint stimulation of the two eyes closely correspond to binocular facilitation psychophysics (Billock et al., 2024). However, for clearly visible stimuli there is little enhancement and the binocular perception is best described by a weighted average of the two eyes. In 1926 Erwin Schrödinger (see MacLeod, 1972 for a detailed description) proposed a particular nonlinear weighted average for the two eyes (Equations 5, 6), to explain some peculiar psychophysical phenomena.


$$\begin{array}{l} \text{Binocular Response}=\mathrm{Ey}e_{1}^{*}(\mathrm{Ey}e_{1}/(\mathrm{Ey}e_{1}+\mathrm{Ey}e_{2}))+\\ \mathrm{Ey}e_{2}^{*}(\mathrm{Ey}e_{2}/(\mathrm{Ey}e_{1}+\mathrm{Ey}e_{2})) \end{array}$$ (5)

or equivalently,


$$\text{Binocular Response}=(\mathrm{Ey}e_{1}^{2}+\mathrm{Ey}e_{2}^{2})/(\mathrm{Ey}e_{1}+\mathrm{Ey}e_{2}).$$ (6)

**Figure 1:**
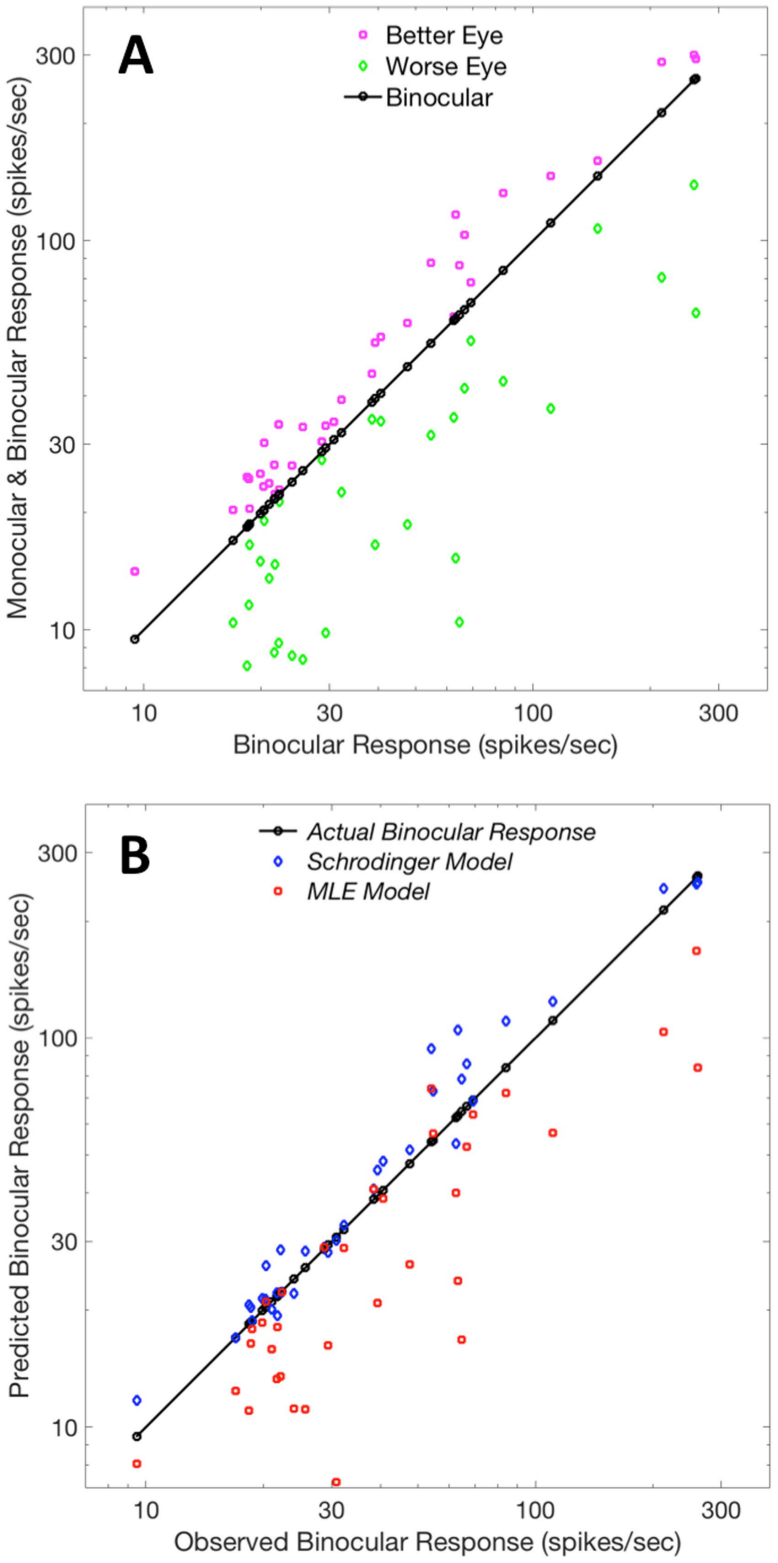
Suppressive binocular neurons in macaque (Case 1). **(A)** 35 mildly suppressive neurons (Dougherty et al., 2019) have binocular responses that lie between the best and worst monocular responses and thus meet MacLeod’s criterion for being some kind of weighted average. **(B)** Plots of modeled response as a function of the observed neural firing rates. The identify line denotes Predicted Response = Neural Response. Schrödinger’s nonlinear weighted average is a better fit than the MLE inverse-variance weighted average, which systematically underestimates the firing rate. Giving both models normalization-fitting-constants drastically improved the Bayesian model, but the Schrödinger model still has the best fit (r^2^_sch_ = 0.96; r^2^_mle_ = 0.76) and needs the least adjustment ‘a’ (a_sch_ = 0.93; a_mle_ = 1.61; vs. 1 for no adjustment). Replotted from calculations in Billock et al. (2024).

Here, *Eye_1_* and *Eye_2_* are the perceived brightness experienced during monocular stimulation. Equation 5 makes explicit that the Schrödinger-model is a weighted average whose weights are determined by the magnitude of the relative monocular responses. Equation 6 makes explicit that this weighted average is nonlinear. Although this model is surprisingly simple, slightly more complicated versions can be derived from gain control theory (Ding and Sperling, 2006; Baker et al., 2012; Baker and Wade, 2017). MacLeod (1972) showed that to fit Schrödinger’s model to psychophysical binocular data, the monocular responses *Eye_1_* and *Eye_2_* need to be nonlinear compressive functions (e.g., logarithms or Naka-Rushton functions) of the contrast of the stimuli, which are common adaptations found in many visual neurons, starting as early as the retina. It then follows that Equations 5, 6 could likely model actual binocular neuron firing rates without further necessary modification, since MacLeod’s modifications would be built into their monocular firing rates. As MacLeod points out, neurons that obey Equations 5, 6 would appear to be suppressive (exhibiting binocular responses that lie below the best monocular responses). It is possible to make this criterion more rigorous: specifically, neurons that average their inputs would appear to be mildly suppressive, exhibiting binocular firing rates that lie between the best and worst monocular responses. We call this the MacLeod Criterion and we call such neurons ‘between’ neurons; they comprise 73% (35/48) of suppressive binocular neurons we have modeled. This criterion, of course, is a necessary but not a sufficient condition for any particular weighted average and applies to both Bayesian MLE averaging and to the Schrödinger model without bias. Billock et al. (2024) examined the 35 ‘between’ binocular neurons from Dougherty et al.’s (2019) study in macaque cortex (see Figure 1A)—they made *Eye_1_* and *Eye_2_* in Equations 5, 6 be the mean monocular stimulation firing rates for each neuron—and found that the Schrödinger model was a much better fit than MLE, even after compensating for the systemic underestimation of neural firing rates by the MLE model (see Figure 1B and Table 1). However, the correlation between the Schrödinger model and the MLE model was high enough (r = 0.88; Table 2) that the Schrödinger model could serve as a Bayesian-like estimator, without the difficulties of implementing Bayesian variance-weighting in neurons. Here we apply this same approach to several different suppressive multisensory neural cell classes examined in various sensory cortices of several species and compare the results to the binocular neurons.

**Table 1:** Fits of Schrödinger and Bayesian MLE models to multimodal neural data^*^.

Cell modality	Animal/Location	Cell#s	Model	Norm (*a* ± se)	r^2^	AICc	Δ_AICc_
Visual-Tactile	Ferret PPr	35	Schröd.	1.005 ± 0.018	0.955	−38.44	0
Visual-Tactile	Ferret PPr	35	MLE	1.028 ± 0.027	0.911	−14.32	24.12
Audio-Tactile	Ferret AAF	14	Schröd.	1.108 ± 0.020	0.983	−31.72	0
Audio-Tactile	Ferret AAF	14	MLE	1.355 ± 0.115	0.652	−23.10	8.62
Audio-Visual	Cat PLLS	7	Schröd.	1.069 ± 0.024	0.988	−9.98	0
Audio-Visual	Cat PLLS	7	MLE	1.310 ± 0.040	0.978	−8.93	1.05
Binocular	Macaque V1	35	Schröd.	0.928 ± 0.023	0.963	174.01	0
Binocular	Macaque V1	35	MLE	1.609 ± 0.017	0.764	238.6	64.59

^*^Multisensory data from this paper, with binocular modeling from Billock et al. (2024) for easy comparison. Both MLE and Schrödinger models, with no fitting constants, yield results that are nearly proportional to the neural firing rate data. The MLE model systematically underestimates combined firing rates and must be compensated for by the normalization factor a. The closer a is to 1.0, the less the model needs to be corrected to make valid predictions. The Schrödinger model always requires less correction and always has a better fit than the Bayesian MLE model regardless of normalization. AICc is the corrected Akaike Information Criterion, a method for comparing models that derives from the information loss that would occur if the model were adopted instead of the actual data (Burnham and Anderson, 2002). The correction is dependent on the number of data points. The actual value and polarity of the AICc are not informative between datasets, but—within a dataset—models with lower values of the AICc are better, and the AICc difference (between two models) for a dataset is key. Δ_AICc_ = AICc (either value)—AICc (lowest value). The evidence against the worst model increases exponentially with Δ_AICc_. A Δ_AICc_ for the worse model of less than two is too weak to argue for excluding the worse model; a difference greater than two but less than seven is increasingly strong evidence against the worse model, and a difference greater than seven is good grounds for excluding the worse model. For Δ_AICc_ greater than ten, the worse model has essentially no support (Burnham and Anderson, 2002). Only for the audio-visual data is the case weak for excluding MLE. All data from Dougherty’s et al. (2019) and Meredith’s (Foxworthy et al., 2013; Meredith et al., 2012; Meredith and Allman, 2015) laboratories.

**Table 2:** Correlations between Schrödinger and Bayesian MLE models for suppressive sensory neurons and percent of neurons showing bimodal variance less than or equal to the lesser of the unimodal variances as predicted by MLE*.

Cell modality	Animal and location	Cell#s	r(Sch, MLE)	% neurons with ≤ variance
Visual-Tactile	Ferret PPr	35	0.993	25.7
Audio-Tactile	Ferret AAF	14	0.834	14.3
Audio-Visual	Cat PLLS	7	0.991	71.4
Binocular	Macaque V1	35	0.883	11.4

* All neural data from Dougherty et al.’s (2019) and Meredith’s (Foxworthy et al., 2013; Meredith et al., 2012; Meredith and Allman, 2015) laboratories. Binocular modeling from Billock et al. (2024) shown for easy comparison to the multisensory data from this paper.

## 2 Theoretical methods

Multisensory neurons that show enhanced responses (relative to their best unisensory responses) have received the lion’s share of attention in multisensory integration. Suppressive neurons do not accord with the more common psychophysical findings for perceptual enhancement (Stein et al., 1996; Gillmeister and Eimer, 2007; Schnupp et al., 2005; To et al. 2011; Billock and Havig, 2018; Billock et al., 2021). One way to account for this finding would be to suppose that suppressive cells provide less stimulation to sensory systems, which seems reasonable, and to note that they are usually much less abundant, which seems truer for some species and brain regions (e.g., in cat superior colliculus) than others (e.g., in ferret and macaque cortex). This paper advances an alternative hypothesis: that a potential neural correlate for Bayesian-like integration has been hiding in plain sight. In MLE, optimal integration requires a weighted sum of the unimodal responses (the weights being determined by inverse relative variances). Leaving aside the actual weights for the moment, a neural weighted average would correspond to neurons whose combined response is less than the best unimodal firing rate but greater than the worst unimodal firing rate. Such cortical suppressive neurons have never been studied in this context, but they are easy to find in the literature on suppressive bimodal cells. This has not been noted before because the usual practice in the multisensory literature is to plot relative enhancement or suppression as a function of the strongest unimodal response. If we examine the bimodal response as a function of both unimodal responses, we can identify such neurons and in some species they are present in large numbers. We test the MLE and Schrödinger models on these data sets. To allow for the possibility that a model might systematically over- or under-estimate the data but still behave proportionally to it, we incorporate a normalization fitting constant ‘*a*’ to quantify the over- or underestimation. Normalization rescaling helps the MLE model more than the Schrödinger model (but the Schrödinger model still has the superior fit). Other than *a* there are no fitting constants—the models are perfectly constrained by the measured firing rate data. This yields the normalized MLE model (Equation 7).


$$\text{MultiSensoryFiringRat}e_{\mathrm{MLE}}=a(\begin{array}{l} \sigma_{2}^{2}\text{Rmod}e_{1}+\\ \sigma_{1}^{2}\text{Rmod}e_{2} \end{array})/(\sigma_{1}^{2}+\sigma_{2}^{2}).$$ (7)

Where *σ_1_^2^ and σ_2_^2^* are the statistical variances associated with the unimodal/unisensory *Rmode_1_* and *Rmode_2_* mean firing rates. Similarly, the normalized Schrödinger model (Equation 8) is


$$\begin{array}{l} \text{MultiSensoryFiringRat}e_{\mathrm{Sch}}=a(\text{Rmod}e_{1}^{2}+\text{Rmod}e_{2}^{2})/\\ (\text{Rmod}e_{1}+\text{Rmod}e_{2}). \end{array}$$ (8)

Here we apply these models to three multisensory cases in addition to the binocular results in the Introduction, which we designate *post hoc* as CASE I.

## 3 Results: modeling neural data

### 3.1 CASE II—visual-tactile suppressive neurons in ferrets

**Figure 2:**
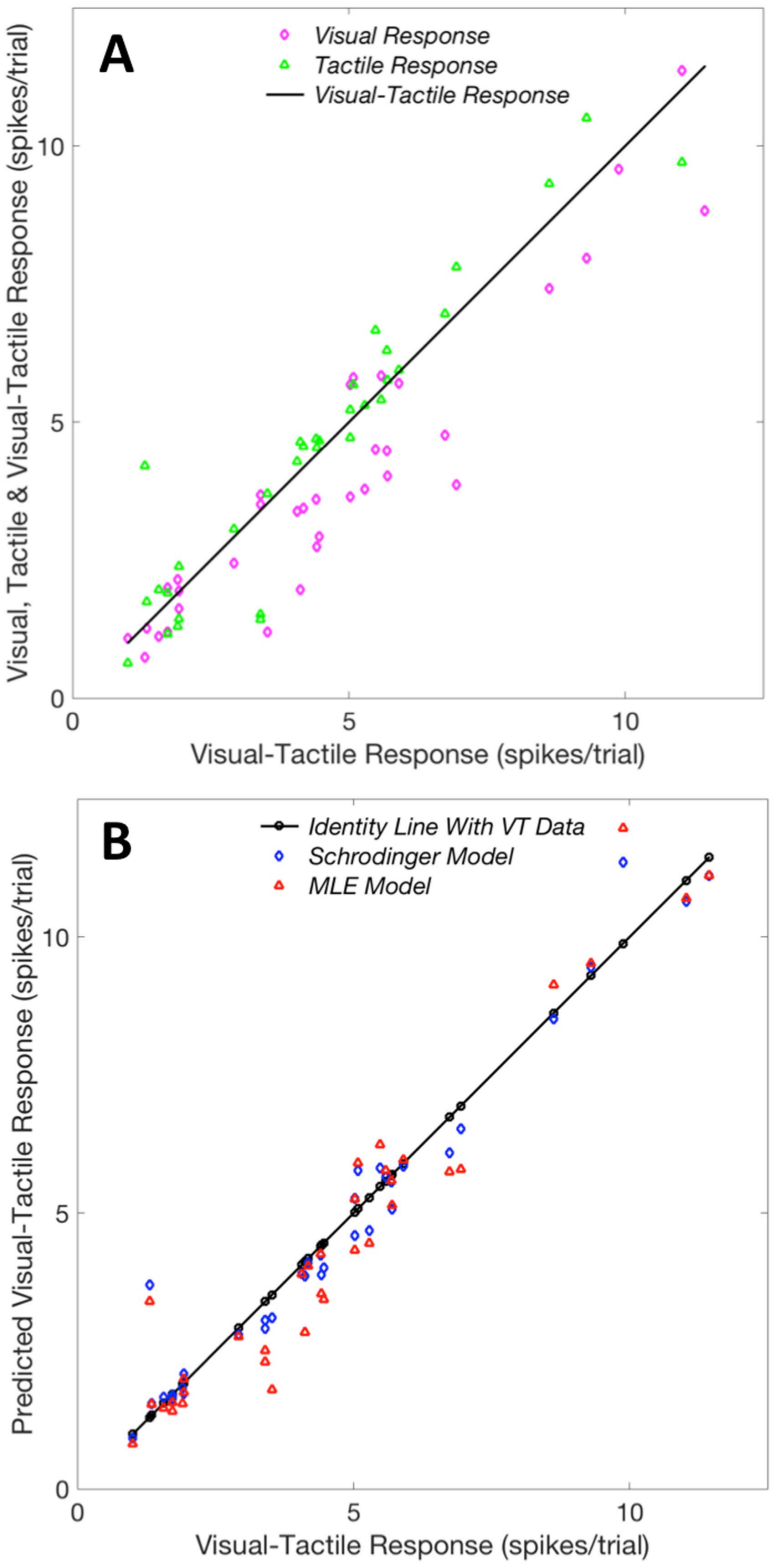
Visual-tactile suppressive neurons in ferrets (Case 2). **(A)** 34 neurons in ferret cortex that have visual-tactile firing rates that lie between the visual and tactile responses (Foxworthy et al., 2013). **(B)** Plots of modeled response as a function of the observed neural firing rates. The identify line denotes Predicted Response = Neural Response. The MLE model requires more normalization adjustment to fit the data and the fit of the Schrödinger model is better regardless (Table 1).

Foxworthy and associates, in Meredith’s laboratory, measured visual, tactile and visual-tactile firing rates in 473 ferret rostral posterior parietal (PPr) cortical neurons (Foxworthy et al., 2013; Foxworthy, 2012). Of these 279 neurons were bimodal for visual and tactile stimuli (Table 3). Of these neurons, 46 were suppressive (had visual-tactile firing rates lower than the stronger of the visual and tactile firings rates) and 35 of those had visual-tactile firing rates between the visual and tactile responses (‘between neurons’), the criterion for potentially being some kind of weighted average (see Figure 2A). Receptive fields were mapped and optimal stimuli were determined with a variety of visual and tactile stimuli. Visual and tactile responses were measured using moving bars of light, and tactile responses were measured using an electrically-driven calibrated filament to displace hairs or indent skin. Visual, tactile and visual-tactile firing rates were measured 50 times each and firing rate means and variances were computed. As with the binocular neurons, the unnormalized MLE model grossly underestimates the actual firing rates, while the unnormalized Schrödinger model is much closer. Even if we compensate for the over/under-estimation of the models (Table 1; Figure 2B), the Schrödinger model (r^2^ = 0.96) has a better fit to the data than the MLE model (r^2^ = 0.91). A model comparison analysis using the corrected Akaike Information Criterion (AICc) yields an AICc difference of 24.12. AICc does not use significance levels but instead compares likelihoods based on information theory (Burnham and Anderson, 2002); a difference greater than about ten is considered overwhelming evidence for the superiority of the better model (in this case Schrödinger’s) over the worse fitting MLE model. By AICc criteria, there is no evidence for the MLE model for this particular visual-tactile dataset. However, both follow the same trend in the firing rate data: the correlation between MLE and the Schrödinger model is 0.99 (Table 2); the Schrödinger model could substitute for the MLE model without any need to employ variance data. Note that this is independent of normalization, which does not alter correlation.

**Table 3:** Relative abundances of multisensory suppressive ‘between’ bimodal cells (neurons that behave like weighted averages of their unisensory responses), suppressive ‘below’ cells (neurons whose combined response is worse than their worst unisensory responses) and the more common ‘facilitatory’ units (units that respond better to combined stimuli than their best unisensory response)*.

Cell modality	Animal and location	Cells	Facilitatory	Between	Below
Visual-Tactile	Ferret PPr	279	233 (83.5%)	35 (12.5%)	11 (3.9%)
Audio-Tactile	Ferret AAF	68	52 (76.5%)	14 (20.6%)	2 (2.9%)
Audio-Visual	Cat PLLS	49	41 (83.7)	7 (14.3)	1 (2.0%)

*Data from Meredith’s (Foxworthy et al., 2013; Meredith et al., 2012; Meredith and Allman, 2015) laboratories.

### 3.2 CASE III—audio-tactile suppressive neurons in ferrets

**Figure 3:**
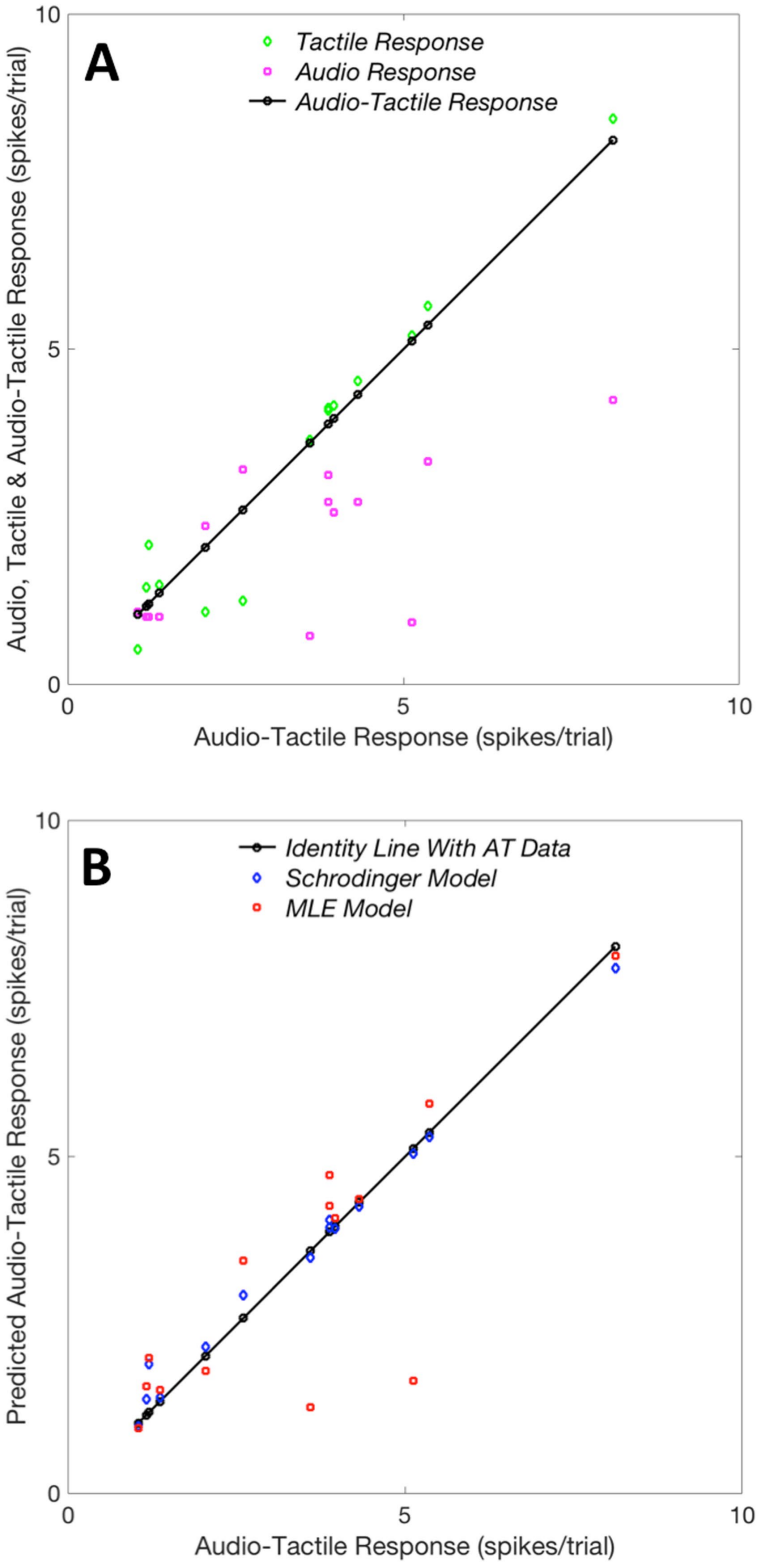
Audio-tactile suppressive neurons in ferrets (Case 3). **(A)** 14 audio-tactile neurons in cat AAF cortex (Meredith and Allman, 2015) whose audio-tactile responses lie between the audio and tactile responses. **(B)** Plots of modeled response as a function of the observed neural firing rates. The Schrödinger model requires less normalization adjustment than the MLE model and yields a superior fit (Table 1).

Meredith et al. (2012) and Meredith and Allman (2015) measured audio, tactile and audio-tactile firing rates and variances for 68 bimodal neurons in ferret cortical area AAF. Of these neurons, 16 were suppressive and 14 of those had audio-tactile responses that lay between the audio and tactile responses (Table 3), the criterion for being some kind of weighted average (plotted in Figure 3A). Audio responses were elicited by electronically generated white noise bursts. Tactile responses were measured using an electrically-driven calibrated filament to displace hairs or indent skin. Audio, tactile and audio-tactile firing rates were each measured 50 times in each neuron to calculate means and variances (Meredith and Allman, 2015). As with the previous two cases, the MLE model grossly underestimates the firing rates, while the Schrödinger model is closer. After normalization (Figure 3B) Schrödinger (r^2^ = 0.98) still provides a much better fit than the MLE model (r^2^ = 0.65). The AICc between the two models is 8.62, a value large enough to consider excluding the MLE model (Burnham and Anderson, 2002). However, the correlation between the two models is 0.83 (Table 3), so if desired the Schrödinger model could likely substitute for MLE in Bayesian modeling without requiring a neural model for weighting variances.

### 3.3 Case IV—audio-visual suppressive neurons in CATS

**Figure 4:**
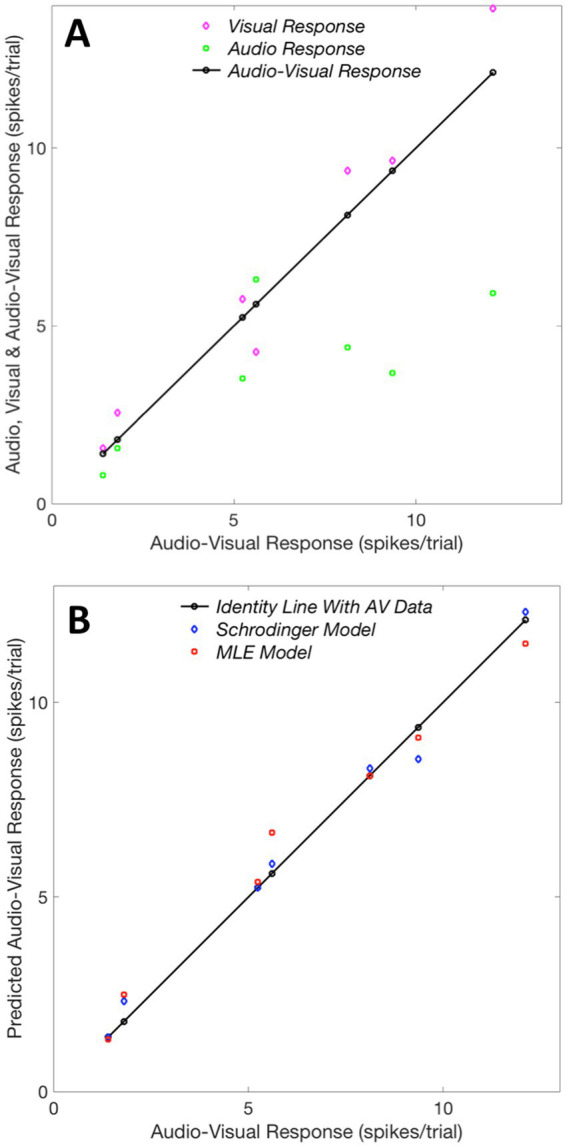
Audio-visual suppressive neurons in cats (Case 4). **(A)** 7 suppressive neurons in cat PLLS cortex (Meredith et al., 2012) that have audio-visual responses that lies between the audio and visual responses. **(B)** Plots of modeled response as a function of the observed neural firing rates. The Schrödinger model is slightly better than the MLE model and requires less adjustment (Table 1).

Meredith et al. (2012) measured audio-visual neurons in cat extrastriate cortex PLLS. Most such neurons are facilitatory, and were modeled in Billock et al. (2021). Eight of 49 bimodal audio-visual neurons were suppressive and 7/8 had audio-visual responses that lie between the audio and visual responses (Figure 4A), the minimal criterion for being some kind of weighted average. Visual stimuli were moving bars of light whose speed, direction, orientation and luminosity produced a maximal response in that particular cell. Similarly, a variety of audio stimuli were tested on each cell and the most effective one was chosen for each neuron for further testing. Each neuron’s firing rate for audio, visual or audio-visual stimulation was measured 23–25 times. As in the other three cases, the unnormalized Schrödinger equation more closely approximates the neural firing rates than the unnormalized MLE model, which always underestimates the data. If we use a normalization constant to negate this advantage of Schrödinger, then the MLE model does almost as well as Schrödinger’s model (Figure 4B) and the correlation between MLE and Schrödinger is very high (r = 0.99; Table 2). A neural model that weights firing rates could substitute in Bayesian theory without needing to weight variances. The AICc difference between the two models is 1.05 (Table 1). The Schrödinger model is slightly superior, but the difference would not exclude the Bayesian model from consideration in this dataset.

### 3.4 What about combined multisensory variance of actual neurons?

A rationale of MLE is that it yields an average with increased reliability (lower variance) relative to its unisensory inputs (Ernst and Banks, 2002; Alais and Burr, 2004; Ernst, 2012; Landy et al., 2011). In theory, an MLE estimator with input variances shown in Equations 2, 3 would have a combined variance (Equation 9) of


$$\sigma_{1,2}^{2}=\sigma_{1}^{2}\sigma_{2}^{2}/(\sigma_{1}^{2}+\sigma_{2}^{2})\leq \min[\sigma_{1}^{2},\sigma_{2}^{2}]$$ (9)

In general, the computed *σ* ^2^*_1,2_* underestimates the actual variances found for multimodal neural firing rates (for audio-tactile and visual-tactile neurons by a factor of 4–5; see Table 4), one can ask a less rigorous question: is the neural multisensory/multimodal variance lower than either unisensory neural variance (is the combined variance even in the right direction)? In general, it is not; only 16/56 multisensory suppressive cells (29%) measured in this study obey the inequality in Equation 9. Interestingly, the audiovisual neurons had the highest proportion of neurons that obey the inequality in Equation 9 and it was the same set of neurons for which the MLE gave its best approximation of the firing rates (Table 1). Finally, one can ask the question: what is the relationship between the firing rate magnitudes and variances? Several studies have found sensory neurons (mostly in V1) that behave in an almost Poisson-like fashion (Tolhurst et al., 1983, Shadlen and Newsome, 1998; Gur et al., 1997), and this has theoretical consequences, some of which have been employed in Bayesian theory (Ma et al., 2006; Beck et al., 2008; Ma and Pouget, 2008). Poisson-like systems should have a strong correlation between mean responses and variances (in a Poisson distribution the mean is equal to the variance). For the neurons studied here, the correlation between the multisensory-mean-firing-rate and the multisensory-firing-rate-variance is generally substantial—averaging 0.80 (Table 5)—suggesting that combined-neural-variances behave in an almost Poisson-like manner rather than combining variances by the MLE rule. This is also in accord with our finding (Table 2) that for most neurons, the multisensory variances fall between the two unisensory variances, the same rule that the firing rate magnitudes follow. That is, if means and variances are well correlated in a population of neurons (as they mostly are in Table 5—less so for visual-tactile neurons) and if means are combined via a nonlinear weighted average (as they are in Table 1), then most of the time the multisensory variance should fall between the unisensory variances, as they do for the mildly suppressive neurons studied here. See Discussion for additional treatment on Poisson-like mechanisms.

**Table 4:** Relationship between MLE-predicted multisensory variance (Equation 9) and actual multisensory variance*.

Multisensory	Cells	a	r
Visual-Tactile	35	0.200	0.917
Audio-Tactile	14	0.273	0.782
Audio-Visual	7	0.674	0.855

*Data from Meredith’s (Foxworthy et al., 2013; Meredith et al., 2012; Meredith and Allman, 2015) laboratories. MLE underestimates the actual variance by up to a factor of 5. Model is MLE Predicted Variance = a*Measured Variance.

**Table 5:** Poisson-like statistics for multisensory suppressive neurons—simple Pearson product correlations between multisensory firing rates (fr) and variances (var)*.

Cell type	Animal and location	Cell#s	Condition	r (fr,var)
Visual-Tactile	Ferret PPr	35	Visual	0.492
Visual-Tactile	Ferret PPr	35	Tactile	0.605
Visual-Tactile	Ferret PPr	35	Visual-Tactile	0.793
Audio-Tactile	Ferret AAF	14	Audio	0.795
Audio-Tactile	Ferret AAF	14	Tactile	0.884
Audio-Tactile	Ferret AAF	14	Audio-Tactile	0.742
Audio-Visual	Cat PLLS	7	Audio	0.699
Audio-Visual	Cat PLLS	7	Visual	0.938
Audio-Visual	Cat PLLS	7	Audio-Visual	0.973
W. Average	All	56×3	All	0.704
W. Average	All	56×2	Monosensory	0.655
W. Average	All	56×1	Multisensory	0.803

*All data from Meredith’s (Foxworthy et al., 2013; Meredith et al., 2012; Meredith and Allman, 2015) laboratory.

## 4 Discussion

### 4.1 Summary and implications

(a) Bayesian Maximum Likelihood Estimation provides a theoretically optimal way to combine multisensory or multimodal sensory information, using inverse-relative-variance-weighted (reliability) averaging, but it is unclear if it is necessary to store variance information over a situationally-dependent temporal window to implement this calculation, particularly in experimental circumstances where reliability is manipulated. It has been argued that inverse variance weighting can be obtained at population-level interactions between neurons, each population encoding only one of the two senses to be combined. This begs the question of what multisensory neurons—particularly ones that seem to compute Bayesian-like weighted averages—are good for. (b) Suppressive neurons can provide a basis for within-neuron weighted averaging in binocular and multisensory integration, because most of these neurons have multimodal responses that lie between the higher and lower unisensory responses. (c) We examined four populations of binocular and multisensory mildly suppressive cells (in 3 different species) that meet the criterion required to be any kind of weighted average. All four cases were approximated by a minor modification of Bayesian reliability-weighted averaging (r^2^: 0.65–0.98), but all four were better approximated by nonlinear magnitude-weighted averaging (r^2^: 0.96–0.99), originally advocated by Schrödinger for binocular perception. Not only is magnitude-weighted averaging a better neural model, but it should be simpler to implement in individual neurons, because it uses real-time information embedded in ongoing firing rates rather than stored/inferred estimates of variances. (d) The multimodal firing rate variances do not, in general, follow the MLE prediction of variance reduction, but instead are well correlated (r = 0.80; Table 5) with multimodal mean firing rates. This suggests that Bayesian-like variance reduction—seen in some (but not all) psychophysical studies—originates at another stage of processing. (e) Moreover, we find a Poisson-like power-law relationship between means and variances of multisensory suppressive neurons (even when responding to simultaneous multisensory inputs) just as there is for unisensory neurons. This implies that suppressive multisensory neural firing rate variances should generally lay between the unisensory variances (just as the mean firing rates do), consistent with our direct findings. (f) It is interesting that the Bayesian model is best as a firing rate predictor for audio-visual combination, since these neurons had the largest percentage of reduced combined firing rate variances, a key MLE attribute. (g) If we neglect the MLE variance-reduction-prediction-discrepancies and only consider mean firing rates, the correlations between the Schrödinger’s nonlinear magnitude weighted averaging and Bayesian MLE reliability-weighted averaging for predicted neural firing rate are high (>0.83; Table 2). This suggests that magnitude-weighting could serve as a surrogate for Bayesian neural calculations (eliding a need for variance information in an early stage of neural processing) and that mildly suppressive binocular/multisensory cells could be early neural correlates of Bayesian-like computation. Schrödinger’s nonlinear means could serve Bayesian ends.

### 4.2 Schrödinger’s equation can be obtained via reciprocal divisive gain control

Schrödinger (1926) was not explicit about how he chose his model for magnitude weighted averaging (he described the model verbally and left the task of writing it out in Equations 5, 6 form to MacLeod, 1972) but the form of Equation 5 suggests that multiplying each monocular response by itself and dividing by the sum was the most straightforward way to construct a nonlinear magnitude-weighted average. However, with the ideas of pooled or reciprocal gain control, neural-inspired models closely resembling Schrödinger’s arose. Ding and Sperling (2006) proposed that the two eyes’ inputs to the cortex should multiplicatively increase their own gain while divisively controlling the gain of the other eye. Let *C_L_* and *C_R_* be contrasts presented to the left and right eyes. The predicted binocular contrast *C_B_* (Equation 10) is


$$C_{B}=C_{L}C_{L}^{\gamma }/(C_{L}^{\gamma }+C_{R}^{\gamma })+C_{R}C_{R}^{\gamma }/(C_{L}^{\gamma }+C_{R}^{\gamma }).$$ (10)

The only difference between this model and the Schrödinger model in Equation 5 is a non-trivial fitting constant *γ*. We considered using this variation to fit firing rates, but the quality of the simpler model’s fits for our data made it unnecessary and unparsimonious. The parameter γ is a fitting constant in this model, but in context—as an exponent—it can also be considered a surrogate for the neural logarithmic nonlinearity in MacLeod’s psychophysical model. For luminance increments it falls in the range of 0.25–0.50, which is strongly compressive like a logarithm. Similarly, Baker et al. (2012) consider a variation of Schrödinger’s equation inspired by the Naka-Rushton function (Equation 11).


$$C_{B}=(C_{L}^{m}+C_{R}^{m})/(C_{L}+C_{R}+S)$$ (11)

Where *m* and *S* are the gain and saturation constants of the Naka-Rushton function. This equation reduces to Schrödinger’s Equation 6 if *m* = 2 and *C_L_* + *C_R_* > > *S* (the high visibility case that MacLeod applied the Schrödinger model to). Note that Equations 10, 11 model psychophysical combinations of monocular stimuli, not combinations of monocular firing rates. These physiological-like adaptions are likely unnecessary for modeling neural firing rates since the nonlinearities are already built into the monocular (or unisensory) firing rates, as MacLeod (1972) anticipated. Baker’s laboratory has tested several variations of Equation 11 on EEG binocular summation (Baker and Wade, 2017), binaural summation (Baker et al., 2020) and in vibro-tactile summation (Wei et al., preprint). An almost identical divisive normalization model for sensory integration neurons has been proposed by Ohshiro et al. (2011, 2017). Ohshiro et al. (2011), although unaware of Schrödinger’s model, even used Schrödinger’s exponent of 2 in the numerator of their model for superior colliculus neurons, but based it on arguments by Heeger (1992), instead of Schrödinger’s rationale.

### 4.3 Schrödinger’s equation can also be obtained from a superior colliculus neural model

Many neurons in superior colliculus can fire super-additively to a combination of visual and auditory stimuli, but their combined response is reduced if descending connections from multisensory cortical areas are blocked. To model these behaviors Rowland et al. (2007) created a biophysical model, which directs ascending sensory input and descending cortical modulation to specific superior colliculus neuron dendritic compartments. The multisensory response (Equation 12) is approximated as


$$MS_{\text{Response}}=\mathrm{\tau ln}{\left[\begin{array}{l} \alpha (V_{u}^{2}+A_{u}^{2}+{\left(V_{d}+A_{d}\right)}^{2})/\\ \left(V_{u}+V_{d}+A_{u}+A_{d}\right) \end{array}\right]}^{+}$$ (12)

Where *τ* and *α* are fitting constants, *V_u_* and *A_u_* are ascending sensory inputs from the visual and auditory receptors, *V_d_* and *A_d_* are visual and auditory top-down inputs from cortical areas AES (anterior ectosylvian sulcus) and rLS (rostral lateral suprasylvian sulcus), and ln[] + is the natural log of positive inputs only. The squaring terms approximate multiplicative interactions driven by synaptic (AMPA and NMDA) connections to the same dendritic compartments. The denominator represents divisive (shunting) inhibitory input to the multisensory neuron from another neuron that simply sums all ascending or descending inputs (e.g., it represents normalization gain control). In this equation the input terms have been rescaled so that an input of one represents a weakly effective stimulus and an input of two represents a very strong stimulus (*Vu* and *Au* are not actual firing rates). This is a model for combining scaled stimuli, not a model for combining monocular firing rates, but if scaled appropriately (using the *τ* and α parameters) it would yield realistic-looking firing rates as its output. Schrödinger’s equation can fall out of the Rowland model in two ways. One is if the model receives two monosensory inputs. For two visual stimuli (*V_1_*, *V_2_*), falling in different portions of a neuron’s receptive field and driving different postsynaptic neural compartments, the output (Equation 13) would look like


$$V_{\text{response}}=\tau *\ln{\left[\alpha (V_{1}^{2}+V_{2}^{2})/(V_{1}+V_{2})\right]}^{+}$$ (13)

Which resembles how MacLeod (1972) would use the Schrödinger model for two monocular inputs to a binocular channel. Alvarado et al. (2008) use it to model two simple visual inputs to the same neuron (for example, *V_1_* and *V_2_* are two bar-shaped objects moving separately through the same receptive field). If *V_1_* and *V_2_* are neural responses to bar stimuli rather than the bars themselves, then a MacLeod-like logarithmic-like nonlinearity is embedded in the firing rates for the individual stimuli, and Alvarado et al. (2008) estimate that the combined visual responses (Equation 14) can be approximated as


$$V_{\text{response}}=(V_{1}^{2}+V_{2}^{2})/(V_{1}+V_{2}).$$ (14)

Alvarado et al.’s (2008) unisensory combined responses generally lie above a simple mean of the individual responses but below the maximum of the responses. As a nonlinear magnitude-weighted average, Equation 14 is well positioned to straddle this divide. The other way a Schrödinger-like equation can fall out of Rowland *et al.*’s model is to use multisensory stimuli but to block descending influences from cortex onto superior colliculus (experimentally this is done by cooling either relevant areas of cortex or their cortico-thalamic projections). If the descending inputs from cortex were blocked, then Equation 12 would lead to Equation 15.


$$VA_{\text{response}}=\mathrm{\tau ln}{\left[\alpha (V^{2}+A^{2})/(V+A)\right]}^{+}$$ (15)

This is very close to Schrödinger’s equation, as we applied it for multisensory inputs to cortical suppressive cells, except we combined firing rates instead of rescaled stimuli, and thus do not require a nonlinear compression. Alvarado *et al*. did not apply the Equation 15 version of Rowland *et al*.’s equation to multisensory responses, but they could have: it would apply mostly to sub-additive Superior Colliculus cells when cortical feedback was deactivated (because suppressive neurons in Superior Colliculus are very rare), and this could be made to work for subadditive cells because the *τ* constant can be used to rescale the output. Similarly, Equations 7, 8 with appropriate values of the normalization constant *a* could be used to model subadditive cells. One nonlinear summation model, Minkowski (vector-like) summation, is used both for modeling cortical multisensory (mostly subadditive neurons; Billock et al., 2021) and as an alternative to Bayesian summation in depth-cue integration (Kemp et al., 2023), but the Minkowski model is probably ill-suited for visual-vestibular neurons, which can show either suppression or partial summation depending on experimental conditions (e.g., cue disagreement; Morgan et al., 2008). (Minkowski’s formula—a modified Pythagorean sum—was designed to sum distances in non-Euclidean spaces, but total distance cannot be less than the largest component distance; Billock et al., 2021.) We do find that both the Schrödinger (Equation 8) and the Bayesian model (Equation 7) with their normalization constants, can work for neurons that have mixed suppressive and subadditive responses depending on conditions (see next section).

### 4.4 Effects of relaxing the MacLeod criterion constraint: can the models be generalized to neurons that are very suppressive or not strictly suppressive?

When fitting rival theoretical models that are both weighted averages (nonlinear-weighted and variance-weighted averages) it makes sense to study neurons whose multisensory firing rates are consistent with weighted averaging. By necessity, as MacLeod (1972) pointed out, these would be categorized as suppressive neurons, because their multimodal response would have to be less than the strongest unisensory response for that cell, and stronger than the weaker unisensory response (what we call the ‘MacLeod criterion’ and what we call ‘between neurons’). This leaves out very strongly suppressive neurons whose multimodal responses lie below both unimodal responses. This does not bias the comparison of the two models, but it does raise the question of what would have happened if we had used both suppressive neuron types. One would expect that including strongly suppressive neurons would weaken the fit of both models, which it does. But it was not obvious to us what effect this would have on which model fit better. We find that: (1) For audio-tactile neurons, including the two ‘below’ neurons has little effect on the fit of the Schrödinger model (r^2^ drops from 0.98 to 0.97), and slightly improves the fit of the MLE model (from 0.65 to 0.68). (2) For the audio-visual neurons, including the one ‘below’ neuron has small effects on the quality of the fits, and the Schrödinger model remains the slightly superior model (r^2^ of 0.99 vs. 0.96). (3) The situation for the visual-tactile neurons is rather different. There are eleven ‘below’ cells here, a rather large number, and most of them are very suppressive (the suppressive multisensory response driving even strong unisensory responses down to about 1–2 spikes/s)—very different than the ‘between’ neurons. The nonlinear regression (Levenberg–Marquardt) algorithm we used on this dataset will not converge for either model, probably because the extreme inhomogeneity of the dataset.

**Figure 5:**
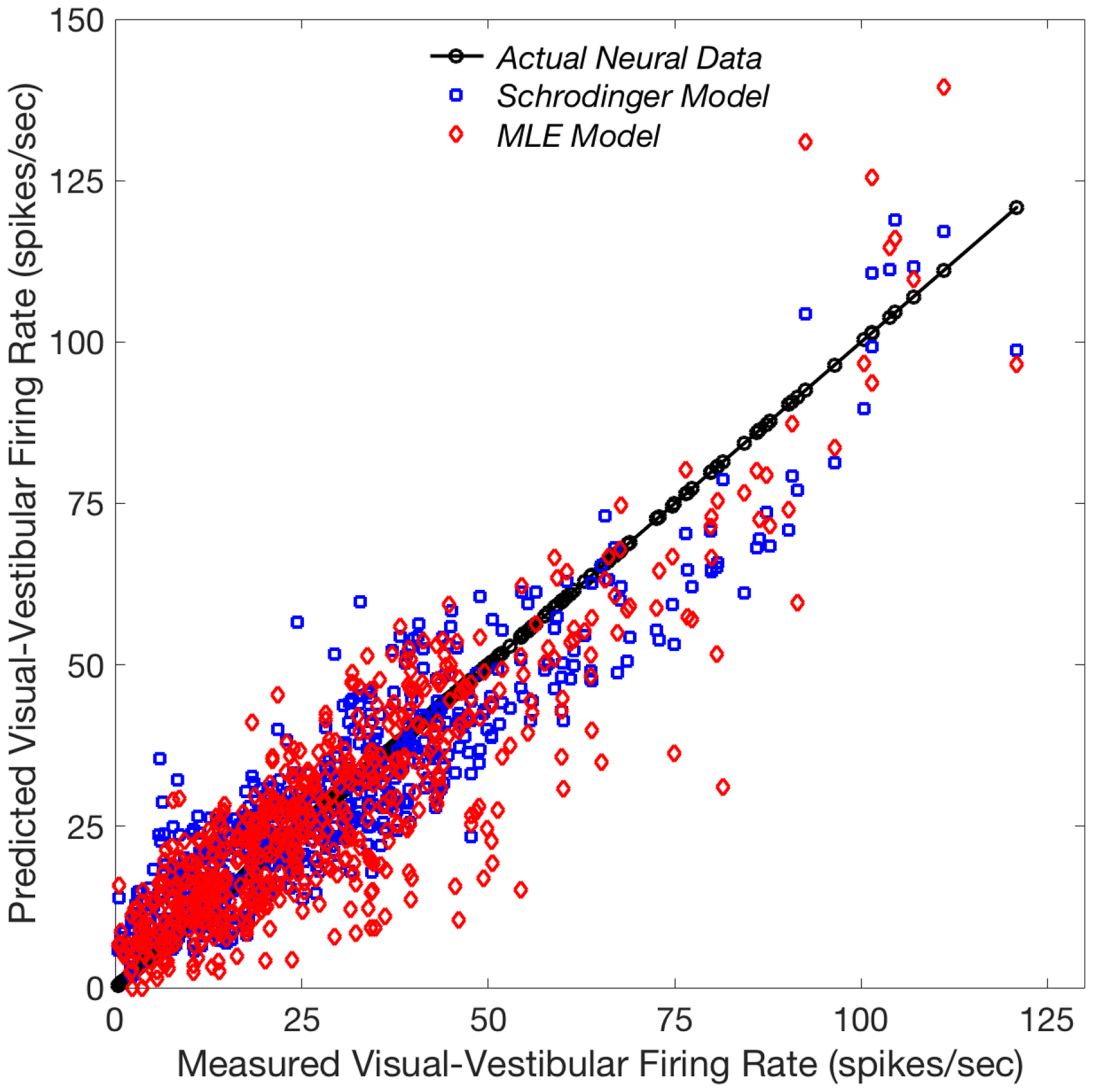
Visual-vestibular conditionally suppressive neurons in monkey. Visual-vestibular neurons can have a variety of preferred visual and vestibular headings. Depending on how these preferences align, a cell can be suppressive at one heading and subbadditive at another. However, both the Schrödinger and MLE models can fit such cells because of the multiplicative constants in Equations 7, 8. Here Billock et al. (2024) modeled 89 macaque MST units at seven heading combinations, taken from Fetsch et al. (2012). The Schrödinger model requires a normalization constant of 1.17 and has an r^2^ of 0.84. The MLE model requires a normalization constant of 1.32 and has an r^2^ of 0.77. Fits are better for the subset of neurons whose heading preferences coincide, and worse for the subset of neurons whose heading preferences conflict, but the Schrödinger model always has a better fit. Figure replotted from Billock et al. (2024); figure uncopyrighted.

Conversely, one can ask if the Schrödinger and MLE models could be applied to neurons that are conditionally but not strictly suppressive. This situation occurs in visual-vestibular neurons that are suppressive for some combinations of visual and vestibular headings, but not others. In theory, the normalization constant ‘*a*’ in Equations 7, 8 would permit this. And in fact, MLE models have been applied to macaque MSTd visual-vestibular neurons in the past (Morgan et al., 2008; Fetsch et al., 2010, 2012) and Morgan et al. (2008) note that the Bayesian weights add to more than 1.0, which could be a sign of nonlinear (compressive) summation, though Morgan *et al*. favor the Bayesian interpretation. Billock (2024) modeled a similar set of 89 macaque MSTd visual-vestibular neurons for 7 different visual-vestibular heading conditions, drawn from Fetsch et al. (2012). The same pattern seen in weighted-average-like suppressive neurons emerges. As expected, both models require a higher normalization constant for these conditionally suppressive neurons than for strictly suppressive ‘between’ neurons. The Schrodinger model is slightly better than the MLE model (r^2^ = 0.84 vs. 0.77; see Figure 5).

### 4.5 Single neuron vs. network implementation of Schrodinger-like models

Attributing cognitive effects to specific neural mechanisms is tricky—e.g., see Bamicha et al. (2025) for a survey of the complications in autism research. Since every data point is the average firing rate of a particular neuron for that set of conditions, we are inclined to view the model as likely instantiated in single neurons. Moreover, there are four separate neural models of how divisive normalization can result in Schrodinger-like models and every one of these has a Naka-Rushton-functional structure, which we know can model individual neural response functions. At least two of these four models have been used to model particular multisensory neural cell types (Alvarado et al., 2008; Ohshiro et al., 2017; Ohshiro et al., 2011; Rowland et al., 2007). So, a single cell implementation is plausible and is supported in our manuscript by the good fits of this model to the sampled neurons whose data we employed. But this does not exclude the possibility that a neural network could also produce an output like a Naka-Rushton function, even if its inputs had a different structure. A nice example of this are computational models in which interacting neural mechanisms with simple logarithmic nonlinear responses can interact to produce a power law response function intrinsic to neither neural mechanism (Billock and Tsou, 2005, 2011). Moreover, if divisive normalization is the computational logic underlying the neural implementation of Schrödinger-like models (e.g., Baker et al., 2012; Baker and Wade, 2017), a neural network model allows for a sampling of divisive inputs over a potentially large spatial range. This would allow models to be tuned for spatial effects, like the Principle of Spatial Coincidence in sensory integration, which tends to be pretty forgiving for relatively small spatial mismatches in stimulus location (e.g., the ventriloquist effect; Stein and Meredith, 1993; Stein et al., 2020).

### 4.6 Some special cases that can implement Bayesian MLE-like averaging and implications for exploitation of Poisson-like neural statistics

The challenge posed by MLE is that to use reliability/variance weighting, the nervous system should in principle keep track of channel variances (Ernst and Banks, 2002), and in the case of psychophysical experiments that manipulate reliability, it should keep track of variance on particular psychophysical time scales. This seems problematic. There are several possible solutions: (1) One possibility (inspired by auditory receptive field multiplication) is to multiply the distributions of channel responses together (which would capture the variances in the calculation *en passant*; Ernst and Banks, 2002). (2) A related model uses a Reichardt-like correlation detector to perform the multiplications and embeds the variances in the calculation by making the receptive field widths in the multiplications inversely proportional to the reliabilities of the channels (Parise and Ernst, 2016). The receptive field size in this model can be thought of as a surrogate for the distribution of channel responses in Ernst and Banks (2002). This clever approach seems difficult to implement physiologically. (3) A related possibility would be to replace temporal variance in MLE models with spatial variance (between similar neighboring neurons), which eliminates a variance storage problem and could be implemented on arbitrary psychophysically-relevant time scales. (4) Another possibility is to take advantage of the vaguely Poisson-like statistics of many sensory neurons (Ma et al., 2006; Beck et al., 2008; Ma and Pouget, 2008). In a Poisson distribution, the means and variances are equal. This is not the case for neurons; for example, Tolhurst et al. (1983) found that variance on average was about three times the mean firing rate (a ‘Fano Factor’ of 3), but a power law fit to the variance/mean-firing-rate data had an exponent of 1.08, which is almost a linear relationship, and in this sense cortical sensory neurons may be said to be Poisson-like. Gur et al. (1997) reviewing several studies, find power law exponents between 1.08 (more linear) and 1.21 (less linear). Applying the Tolhurst-Gur power law relationship to multisensory neurons we obtain exponents for the unisensory variance prediction (Table 6) that range from 0.98 (quite linear) to 1.75 (very nonlinear). The use of simple correlation between firing rate means and variances may be a useful gauge of Poisson-like behavior (for exponents relatively close to 1) because the discrepancy between the relative magnitudes of firing rate and variances can be altered by some experimental designs (Gur et al., 1997), but this would not change the correlation, if mean and variance remain proportional. For a true Poisson process the correlation should approach 1.0; for Gur et al.’s data the correlation was 0.76. This resembles the average correlation of 0.71 for our three multisensory suppressive neural cases (Table 5) for both single modality and bimodal inputs, and suggests that Gur et al.’s results may extend to multiple sensory cortices. It also suggests that an MLE calculation based on *unisensory* channel firing rates and their Poisson-like relationship to variability (correlation of 0.66 for our data; Table 5) might roughly implement an approximation of MLE variance weighting. Some models of attractor neural networks using Poisson-like input neurons have arrived at Bayesian-like decision making (Ma et al., 2006; Beck et al., 2008; Ma and Pouget, 2008). It is not clear from those studies whether a correlation of 0.66 is Poisson-enough to get a sufficiently Bayesian outcome [but see the Supplement to Ma et al. (2006) for some special cases]. In any case, this Poisson-adjacent statistical data might inform such a calculation.

**Table 6:** Poisson-like firing rate statistics (Variance = a*Mean^n^ fits) for three populations of multisensory suppressive neurons*.

System/Animal	Condition	Neurons	Fano factor	a	n	r
Vis.-Tact. Ferret	Visual	35	4.57	1.33	1.75	0.62
Vis.-Tact. Ferret	Tactile	35	2.40	0.69	1.65	0.82
Vis.-Tact. Ferret	Vis.-Tact.	35	4.72	1.78	1.65	0.49
Aud.-Tact. Ferret	Audio	14	1.38	0.64	1.73	0.82
Aud.-Tact. Ferret	Tactile	14	1.90	2.18	0.92	0.89
Aud.-Tact. Ferret	Aud.-Tact.	14	1.72	0.95	1.38	0.76
Aud.-Vis. Cat	Audio	7	1.82	1.88	0.98	0.70
Aud.-Vis. Cat	Visual	7	1.39	1.00	1.12	0.94
Aud.-Vis. Cat	Audio-Visual	7	0.86	0.88	0.99	0.97

*See Gur et al. (1997) for a discussion of this model as used in purely visual cortical neurons. The ratio of variance to mean firing rate is widely known in the literature as the ‘Fano Factor’ and is most often greater than 1.0. The audiovisual combination data comes closest to true Poisson-like behavior; the Fano Factor is close to 1.0 for the audiovisual combination, and the power law exponent is very close to 1.0—this is also the only dataset that shows a majority of neurons (see Table 2) that reduce variance for a multisensory combination. All data from Meredith’s laboratory (Foxworthy et al., 2013; Meredith et al., 2012; Meredith and Allman, 2015).

### 4.7 Psychophysical considerations: considering MLE and its alternative

Here we found that although the Schrödinger model is a better model for combined firing rates (for every data set and both criteria), the r^2^ values between the neural firing rates and the MLE model in Table 1 ranges from a modest 0.65 to a strong 0.98. This suggests that if we compensate for the near-constant Bayesian underestimation, MLE (Equation 7) would often be adequate to predict multisensory firing rates (although for some models an estimate proportional to firing rate might suffice). This is interesting because there is a substantial literature suggesting that psychophysics are often consistent with the Bayesian prediction, both in magnitude and variance. However, although the neural firing rates can be treated as a MLE estimator for firing rate magnitude, their combined variances are not consistently lower than the firing rate variance for a single mode. Indeed, the combined firing rate variances are fairly well correlated with the combined mean firing rates (r = 0.80; Table 5). One way to square this with psychophysics is to posit that psychophysics will likely be influenced by subsequent post-processing based on interactions of many neurons and that the effects of these interactions on psychophysical variability may not be simpatico with individual bimodal neurons at the initial stage of the processing. However, cue weighting that does not result in variability reduction is not unheard of, even in psychophysics, as are data that suggest non-Bayesian cue weighting. Rahnev and Denison (2018) reviewed deviations from Bayesian optimality in many perceptual domains. Rosas et al. (2005) found (for combinations of visual and haptic texture information) that in less than 20% of measured combinations did the psychophysical data follow MLE optimal cue combination. Coincidentally, this resembles our finding (Table 2) that 22% of multisensory and multimodal suppressive neurons show variability reduction for the combined case. In some cases the combined variability in Rosas *et al*.’s data lay between the unisensory variances, which corresponds with the majority of the neurons we studied. Rosas et al. (2005) note that they found these cases puzzling, since the observers would have been better off ignoring the weaker cue. This is an analog of Fechner’s binocular paradox—if one eye views an image through a dark filter, the image is brighter if the impoverished eye is shut, even though the total stimulation to the binocular system is reduced. Fechner’s paradox is exactly the situation that Schrödinger’s nonlinear magnitude-weighted average model was intended to address; impoverished cues get small but non-negligible inputs to the averaged response. For a related problem, Kemp et al. (2023) and Domini (2022) compared MLE and vector models of depth cue integration and found that vector combination (which is closely related to Schrödinger’s model) works better than MLE for depth. Similarly, Oruc et al. (2003) reported that only 3 of 8 observers were completely consistent with MLE-like optimal cue combination of visual perspective and texture cues in a slant judgment task. Meijer et al. (2019) reported cases of audiovisual integration in which the required cue weightings were not consistent with MLE, and Butler et al. (2010) reported similar mis-weighting for visual-vestibular integration. It would be interesting to try a Schrödinger-like nonlinear weighting model (for examples see Equations 10–13) on some of these psychophysical datasets.

### 4.8 Comparing nonlinear and Bayesian neural weighting models

There are three interesting ways to think about a relationship between nonlinear and Bayesian-like weighting in neurons. First, if we speak of reliability-weighting, *it is possible to think of Schrödinger’s nonlinear magnitude weighting as a type of reliability-weighting—when dealing with relatively small numbers of action potentials in a decision window, quantity has a quality all its own*. See Rosas and Wichmann (2011) for a intriguing discussion of the non-optimality of variance for reliability weighting and see Kemp et al. (2023) and Domini (2022), whose vector-cue-combination model seeks the most stable interpretation of cue combination rather than the most probable interpretation. Baker and Wade (2017) have proposed a variant of the Schrödinger model for optimal integration of neural signals. Second, in cases where an increase or decrease in cue reliability results in a corresponding increase or decrease in the neural firing rate (which is known to happen when visual coherence is manipulated in visual-vestibular integration; Morgan et al., 2008; Fetsch et al., 2012), Schrödinger’s nonlinear magnitude-weighted model will result in an automatic reweighting of that channel in the bimodal response (as is also found psychophysically). Third, the data at the bimodal cell level studied here suggest that the actual implementation for individual neurons is more like a magnitude-weighted averaging (Table 1), but that Schrödinger’s magnitude-weighted averaging approximates Bayesian MLE combination well enough to substitute for it in a pseudo-Bayesian theory, with correlations between 0.83 and 0.99 (Table 2). And one limitation of implementing MLE might be that neural divisive normalization and output nonlinearities could distort the linear readout that MLE presumes. All these raise the possibility that—even if there was evolutionary pressure towards a reliability-weighted system—an easier-to-implement nonlinear magnitude weighted approximation may have evolved instead, at least in the early stages of neural processing. Baker and Wade (2017) note some mathematical resemblances between MLE and their version of the Schrödinger equation (which they derived using divisive gain control); they speculate that that one of this nonlinearity’s evolutionary advantages is that it allows it to produce a result similar to Bayesian reliability weighting. As Scarfe (2022) has pointed out, rival cue combination models are often highly correlated. This might be expected because they are both being fitted to the same data. However, our models, except for a normalization constant (which does not affect the correlation), are completely constrained by the data; the models are naturally coincident, and this has a potential evolutionary interpretation. In theory, evolution can be modeled in a fitness landscape where the optimal solution is expressed as a global maximum in the landscape. However, there is no evidence that evolution always finds a global maximum, but rather often settles for a good enough solution, especially if it is less costly in other ways (it settles on a local maximum rather than a global maximum). Even if the optimal solution to sensory cue combination were an inverse relative variance-weighting, given difficulties in sensory systems extracting variances, a simple nonlinear weighting that is well correlated with variance weighting might have sufficed, at least in early stages of neural processing.

### 4.9 No clear distinction between multisensory and multimodal integration in cortex

The distinction between multisensory and multimodal integration is sometimes nebulous in perception and in neural systems. Consider the rattlesnake optic tectum, which combines visual information from the eyes and infrared information from the facial pits adjacent to the eyes (Newman and Hartline, 1981, 1982). This is considered sensory integration mostly because the information originates in separate optic organs. But it might be valid to consider it visual binding; two optical modalities merging into a coherent percept (Billock and Tsou, 2014). For that matter, if the infrared information had been transduced by the retinas, we could consider it a form of color vision, not too different than the interactions between other wavelength-tuned mechanisms (Billock and Tsou, 2005; Billock et al., 2025). Here we applied a model from binocular vision to multisensory integration. Elsewhere we have applied a model from multisensory integration to a different problem in binocular vision and to a phenomenon from color vision (Billock and Havig, 2018; Billock et al., 2025). All told, there are three generic models that work particularly well for both sensory integration and multimodal integration and each of these models is associated with a distinct neural mechanism. The neural mechanisms considered here apply to both binocular and multisensory integration, provides a role for suppressive neurons, follows Schrödinger’s nonlinear magnitude weighted averaging model, and seem to imperfectly mimic Bayesian MLE-like reliability weighting. The other two models—gated amplification and vector-like (Minkowski) summation—have neural substrates in gated binocular neurons/subthreshold multisensory neurons (Billock and Havig, 2018; Billock et al., 2024) and in cortical bimodal neurons (Billock et al., 2021) respectively. In each case, the statistical properties and scaling behaviors of neural and perceptual systems seem in close correspondence (Billock and Havig, 2018; Billock et al., 2021). It may be that the nervous system evolved a small repertoire of combinatory mechanisms that are used and reused for multisensory and multimodal integration alike.

### 4.10 Limitations of the study and potential for future research

There are several primary limitations of this study and there are knowledge gaps that would be useful to fill: (1) We used the standard inverse-relative variance-weighted Maximum Likelihood Model as a representative form of Bayesian cue combination. We did so because most psychophysical cue combination studies used inverse-relative-variance weighting and we wanted neural information combination models to be roughly comparable to those psychophysical studies. The standard Maximum Likelihood Estimation model can be derived from a Bayesian cue combination model with a flat prior, and assumes Gaussian statistics (Ernst, 2012; but this is not the only way to derive MLE—see Young et al., 1993). We know from the statistics in Table 3 that the means and variances are not independent like Gaussian statistics, but they are not as correlated as Poisson statistics. It might be that a different set of statistical assumptions would lead to an improved neural Bayesian cue combination model, in the cases where Schrödinger’s model currently fits noticeably better. (2) It is also possible that a different kind of Bayesian modeling might do better in some respects than MLE. In a fascinating analysis, Scarfe (2022) points out that a model in which the multisensory system chooses one of two cues and switches between them rapidly and spontaneously (with switch probabilities proportional to cue strengths) can on average produce results identical to Equation 4, but with a combined variance that is larger than would be predicted by Equation 9. This would raise the percentage of neurons with combined firing rate variances closer to one Bayesian-like prediction. However, this probabilistic-like switching does not seem a likely behavior within single bimodal neurons and therefore we did not pursue this line of investigation. (3) We used the spike rate variances of each individual neuron as the variances for the neural MLE models. But we have no way of knowing if the trial-wise spike-count variances are good proxies for the estimator variances that MLE requires. Perhaps a neural MLE model might make use of spike rate variances derived from a neighborhood pool of neurons, over a particular temporal window. Similarly, MLE assumes independence of the sensory drives being combined, but we have no data on whether the two drives exhibit shared gain, or in-common noise, or slow drifts that would undermine conditional independence. (4) We have not modeled psychophysical studies in parallel with these neural studies. Some psychophysical studies show deviations from MLE models (Butler et al., 2010; Kemp et al., 2023; Meijer et al., 2019; Rahnev and Denson, 2018; Rosas et al., 2005, Oruc et al., 2003), but we have not shown that Schrödinger’s model would work well in multisensory psychophysics, although we do know of several binocular psychophysical studies where Schrödinger’s model and its variations work well (MacLeod, 1972; Ding and Sperling, 2006; Baker et al., 2012). (5) It is interesting that the multisensory data set that Schrödinger beat MLE the most by (the visual-tactile neurons) was the dataset with the biggest Fano factors (i.e., the noisiest data, based on the ratio of the variance to the mean firing rates). It is possible that the nonlinearly weighted Schrödinger model is particularly good at modeling neurons which have noisy/‘bursty’ unisensory inputs. It would be useful if more suppressive multisensory neural datasets were available to model. (6) Finally, there are nuances in psychophysical cue combinations (first studied in visual multimodal integration; Young et al., 1993; Landy et al., 1995) that would be difficult or problematic to fully implement in individual multisensory neurons. It is possible to implement cue combination in neural networks using population-based coding (Ma et al., 2006; Ma and Pouget, 2008; Beck et al., 2008; Sohn and Narain, 2021), but that would not strictly require the existence of multisensory neurons at all, let alone the multisensory neurons in Figures 2–4 that mimic Bayesian-like combination. This approach—if taken to its logical extreme—begs the question of what multisensory cortical cells are even for. The literature is full of multiple types of these multisensory cortical neurons and each neural type has a behavior that mimics a particular psychophysical finding. Elsewhere we have shown that excitatory multisensory bimodal cortical neurons mirror the scaling properties of multisensory detection threshold data (Billock et al., 2021), and that modulated unisensory cortical neurons (subthreshold multisensory neurons) mirror the scaling properties of multisensory amplification of brightness and loudness (Billock and Havig, 2018). Similar findings occur for predicting monocular and binocular contrast sensitivity from neural responses of ordinary binocular and gated binocular neurons (Billock et al., 2024). It would not be unreasonable if neurons that mimic Bayesian-like neural response combination were involved in Bayesian-like cue combination, but the variance reduction seen in some psychophysical studies would not arise in those neurons. To the extent that variance reduction holds true psychophysically, its neural correlates must occur at a different stage than the multisensory neurons we studied or perhaps emerge from network-level interactions.

### 4.11 Preliminary conclusions

Because we find that the Schrödinger model outperforms the Bayesian model for five different neural firing rate datasets (the three multisensory suppressive neuron datasets in the Results section; the binocular suppressive neurons from Billock et al. (2024), discussed in the Introduction; and the conditionally suppressive visual-vestibular neurons from Billock (2024) discussed in Section 4.4), we posit that the Schrödinger model corresponds to a generic neural mechanism for averaging multiple neural inputs. The plausibility of this conclusion is strengthened by three different laboratories having independently derived Schrödinger-type models from basic neural principles (Ding and Sperling, 2006; Rowland et al., 2007; Baker et al., 2012) and by subsequent work from those laboratories. Because the Schrödinger model produces mean firing rates that are correlated with the less well-fitting Bayesian MLE model, we suggest examination of psychophysical data that has been modeled with MLE models (including datasets that do not show variance reduction) to see how Schrödinger-like models might fare for the same datasets.

## Data Availability

The original contributions presented in the study are included in the article/supplementary material, further inquiries can be directed to the corresponding author.
